# Imaging Synaptic Density: The Next Holy Grail of Neuroscience?

**DOI:** 10.3389/fnins.2022.796129

**Published:** 2022-03-25

**Authors:** Maria Elisa Serrano, Eugene Kim, Marija M. Petrinovic, Federico Turkheimer, Diana Cash

**Affiliations:** ^1^Department of Neuroimaging, The BRAIN Centre, Institute of Psychiatry, Psychology and Neuroscience, King’s College London, London, United Kingdom; ^2^Department of Neuroimaging, Institute of Psychiatry, Psychology and Neuroscience, Kings College London, London, United Kingdom; ^3^Department of Forensic and Neurodevelopmental Sciences, Institute of Psychiatry, Psychology and Neuroscience, King’s College London, London, United Kingdom; ^4^MRC Centre for Neurodevelopmental Disorders, King’s College London, London, United Kingdom

**Keywords:** electron microscopy, immunohistochemistry, SV2A, PET, GluCEST, synaptic density

## Abstract

The brain is the central and most complex organ in the nervous system, comprising billions of neurons that constantly communicate through trillions of connections called synapses. Despite being formed mainly during prenatal and early postnatal development, synapses are continually refined and eliminated throughout life via complicated and hitherto incompletely understood mechanisms. Failure to correctly regulate the numbers and distribution of synapses has been associated with many neurological and psychiatric disorders, including autism, epilepsy, Alzheimer’s disease, and schizophrenia. Therefore, measurements of brain synaptic density, as well as early detection of synaptic dysfunction, are essential for understanding normal and abnormal brain development. To date, multiple synaptic density markers have been proposed and investigated in experimental models of brain disorders. The majority of the gold standard methodologies (e.g., electron microscopy or immunohistochemistry) visualize synapses or measure changes in pre- and postsynaptic proteins *ex vivo*. However, the invasive nature of these classic methodologies precludes their use in living organisms. The recent development of positron emission tomography (PET) tracers [such as (^18^F)UCB-H or (^11^C)UCB-J] that bind to a putative synaptic density marker, the synaptic vesicle 2A (SV2A) protein, is heralding a likely paradigm shift in detecting synaptic alterations in patients. Despite their limited specificity, novel, non-invasive magnetic resonance (MR)-based methods also show promise in inferring synaptic information by linking to glutamate neurotransmission. Although promising, all these methods entail various advantages and limitations that must be addressed before becoming part of routine clinical practice. In this review, we summarize and discuss current *ex vivo* and *in vivo* methods of quantifying synaptic density, including an evaluation of their reliability and experimental utility. We conclude with a critical assessment of challenges that need to be overcome before successfully employing synaptic density biomarkers as diagnostic and/or prognostic tools in the study of neurological and neuropsychiatric disorders.

## Introduction

**FIGURE 1:**
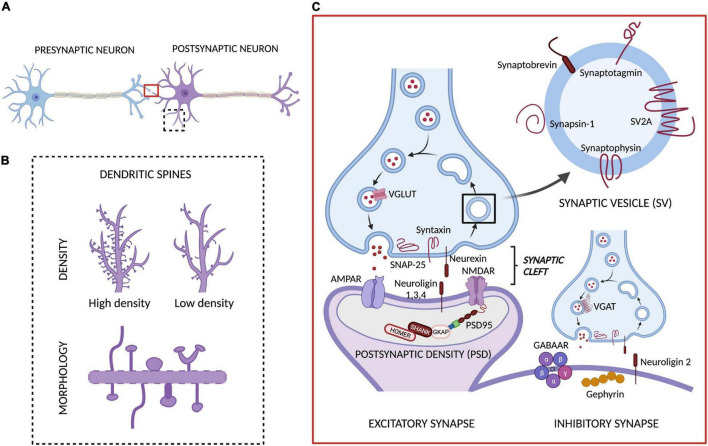
Chemical synapse and targets for measuring synaptic density. **(A)** The main components of the bipartite synapse: a presynaptic and a postsynaptic neuron, separated by a synaptic cleft. In this synapse we have highlighted the two main targets involved in the methods currently available to quantify or assess synaptic density levels: **(B)** the dendritic spines (whose density and morphology are typically evaluated with different *ex vivo* methods) and **(C)** the proteins involved in synaptic transmission, including synaptic vesicle (SV), presynaptic and postsynaptic proteins present in excitatory and inhibitory synapses (which are quantified through *ex vivo* and *in vivo* methods). Adapted from “Synaptic Cleft (Horizontal),” by BioRender.com (2021). Retrieved from https://app.biorender.com/biorender-templates.

Neurons are the fundamental units of the brain through which all bodily functions are coordinated. To achieve this level of global control, neurons communicate with each other through a network of *synapses*: the fundamental information processing units in the brain, which receive, process and transmit all the information (Stampanoni Bassi et al., 2019). In the central nervous system (CNS), the synapses can be classified as different types depending on: (1) the part of the presynaptic neuron connected to the postsynaptic neuron (axoaxonic, axosomatic, and axodendritic synapses), (2) the mechanism of information transmission between neurons (electrical or chemical synapses) or, in the case of chemical synapses, (3) the type of neurotransmitter involved in this transmission (excitatory and inhibitory synapses) (Torres and Varona, 2012; Caire et al., 2021). For the purpose of this review, we will exclusively focus on the *(bipartite) chemical synapse* — when a chemical (*neurotransmitter*) transduces an electrical impulse into chemical information (*receptor binding*) via a gap between a presynaptic and a postsynaptic neuron (*synaptic cleft*). This type of synapse is, by far, the most utilized in the CNS and, therefore, the main target of the existing methods to visualize or quantify synapses. Currently, these methods rely on detecting any one of the elements present in the presynaptic or the postsynaptic neuron, be it a morphological feature such as a change in dendritic spines (small dynamic protrusions located along the dendrites), or variations in the expression of a specific protein from the synaptic vesicle cycle or the postsynaptic density (PSD, an electron-dense structure located beneath the postsynaptic neuron’s membrane, usually at the tip of the dendritic spine) (see Figure 1).

Recently, the interest in quantifying synaptic density has significantly increased. However, the concept of *synaptic density* has never been formally defined, which is reflected in the difficulties faced by researchers when measuring this parameter, and the controversy interpreting the results obtained with different methods. To understand the implications of measuring synaptic density, we should first consider how the number of synapses in the brain changes throughout life. During embryonic, neonatal, and adolescent brain development, new synapses are continually formed through a process known as *synaptogenesis*, after which they are either strengthened or, when no longer useful, eliminated through *synaptic pruning* (Oberman and Pascual-Leone, 2013; Power and Schlaggar, 2017). Although some synaptogenesis continues throughout life, the main assemblage of neural connections in an individual is likely completed by the end of adolescence, and this underpins the correct functioning of the nervous system (Peter, 1979; Paolicelli et al., 2011; Sakai, 2020). Thus, the concept of synaptic density is used to mean the net number of surviving synapses which changes very little in adulthood, except due to the influence of neurodevelopmental abnormalities, or during some neurodegenerative disorders (Lepeta et al., 2016).

The importance of correct synaptic organization is highlighted by emerging evidence that early life problems with either synaptogenesis or synaptic pruning may underpin many disorders of the nervous system (Cardozo et al., 2019). This includes disorders that are considered neurodevelopmental in origin (such as autism or schizophrenia), as well as psychiatric disorders (such as depression), and even neurodegenerative diseases that emerge later in life, despite having a proposed lengthy prodromal phase [such as Alzheimer’s disease (AD)]. Moreover, synapse loss is a hallmark of disorders that are traumatic in nature — such as stroke or brain injury — and even epilepsy (Murphy and Corbett, 2009; Rabiner, 2018; Jamjoom et al., 2021). More research in this field is needed to fully understand the importance of synapses, their number and organization, for proper function of the healthy CNS. What is unquestionable is that synaptic organization is dynamic and delicate, and that even small alterations at any developmental point may lead to profound imbalances and a variety of symptoms to sufferers. Research into autism, for example, has highlighted a possible relationship between abnormal brain development, symptomatology, and an excess of synapses via inadequate synaptic pruning (Neniskyte and Gross, 2017). Schizophrenia, on the other hand may be linked to excessive synaptic pruning within the prefrontal cortical brain circuitry (Sekar et al., 2016; Obi-Nagata et al., 2019; Keshavan et al., 2020; Germann et al., 2021). Synaptic loss in cortical and hippocampal brain areas in AD is well documented and robustly linked to cognitive symptoms of memory loss, and deficits in attention and thought organization (Selkoe, 2002; Knobloch and Mansuy, 2008; Scheff et al., 2014; Jackson et al., 2019; Colom-Cadena et al., 2020). This line of research resulted in the emergence of a novel theory positing that many, if not all neurodegenerative disorders are likely to be *synaptopathies*: disorders featuring disturbances in neuronal connectivity, in which the loss of synapses often features earlier than any other core symptoms (Grant, 2012; Lepeta et al., 2016; Luo et al., 2018). For example, synaptic pathology is present even in early stages of Huntington’s and Parkinson’s diseases (Li et al., 2003; Imbriani et al., 2018), as well as in related neurodegenerative disorders such as amyotrophic lateral sclerosis (ALS) and frontotemporal dementia (Taoufik et al., 2018; Fogarty, 2019).

Given the importance of appropriate synaptic numbers and connections in the living brain, accurate ways to determine their density are needed for research and diagnosis of psychiatric and neurological conditions. However, counting synapses and measuring their density is not a novel concept. Indeed, a plethora of validated methods exist, albeit the majority are performed *ex vivo*. The measurement of synaptic density *in vivo* is substantially more challenging and novel imaging methods are attempting to achieve this.

In this review, we first briefly summarize the existing *ex vivo* methods for measuring synaptic density and comment on their applications in neuroscience. Although all the methods and concepts outlined can and have been used in clinical as well as in preclinical research, here we focus on examples from preclinical (non-human) studies since the majority of *ex vivo* evaluations tend to be undertaken in experimental animal models. Furthermore, we provide a critique of these approaches, showcasing their strengths and weaknesses. We then discuss the current and emerging methods that attempt to measure synapses *in vivo*, and we comment on their utility in different settings, as well as the degree of their validation against gold standards. The overall aim of this review is to underline the importance of studying synaptic density in both the healthy brain and in neurological and neuropsychiatric disorders, as well as to help the reader select the most suitable tools with which to measure synaptic density for their own research.

## Measuring Synaptic Density *ex vivo*

To date, the gold standard methods for measuring synaptic density are conducted *ex vivo* (i.e., in post-mortem tissues) and involve either high resolution electron microscopy (EM), immunohistochemistry (IHC) or both. Although these methods preclude the longitudinal evaluation of the animals or patients under assessment — therefore unable to follow the development of a pathology or the response to treatment — they nevertheless tend to be more direct and specific, allowing more detailed analyses than *in vivo* methods due to their superior spatial resolution. These techniques focus predominantly on one of the three main aspects of the synapse: structure and spatial organization (EM), the morphology and density of dendritic spines (EM and histology), and the expression of proteins in the pre- and postsynaptic neurons, especially in the presynaptic active zone and the postsynaptic density (PSD) area (immuno-EM and IHC).

This section covers the assessment of synapses by EM and IHC, including some examples of applications in preclinical research. Although alternative, non-imaging *ex vivo* methods to measure parameters related to synaptic density exist (such as Western Blot and proteomics), they are beyond the scope of this review and are covered in other reviews such as (Ippolito and Eroglu, 2010; Patrizio and Specht, 2016).

### Electron Microscopy

**FIGURE 2:**
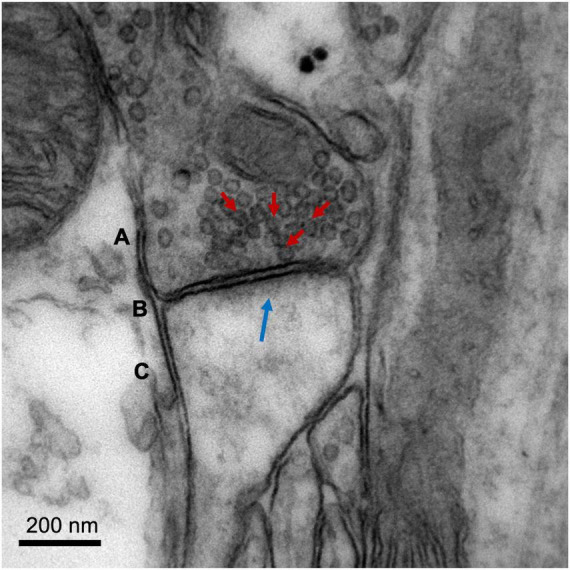
Image of a cortical glutamatergic synapse of an adult C57BL/6 mouse. The image was obtained on a Jeol 1010 transmission electron microscope (Jeol, Tokyo, Japan) at 80,000× magnification. (A) Presynaptic neuron, with synaptic vesicles indicated with red arrows. (B) Synaptic cleft. (C) Dendritic spine of a postsynaptic neuron, with postsynaptic density (electron-dense zone juxtaposed to the postsynaptic membrane) indicated with a blue arrow. Image courtesy of Nuria García Font, see García-Font et al. (2019) for more information about the methodology.

Electron microscopy (EM) is the gold standard method to quantify changes in synaptic density, being the only technique that allows direct visualization of the synapse and its molecular organization due to its ultra-high spatial resolution (nanometer range) (Figure 2). Successful applications of EM have led to major breakthroughs in the field of neuroscience, such as the discovery of synaptic vesicles (Wells, 2005), the nature of dendrite spines as sites of synaptic contact (Gray, 1959), and the existence of cells other than neurons (such as astrocytes) involved in synaptic transmission (tripartite synapse) (Ventura and Harris, 1999).

There are two main types of electron microscopes, both employed to assess synaptic density: transmission (TEM) and scanning (SEM) electron microscopes. Although both use an electron source, they provide different information about the sample and are used for distinct purposes. In a TEM, the electrons pass through the tissue sample before they are collected. This system offers high spatial resolution (<50 pm) and provides valuable information about the inner structure of the synapse (e.g., area of active zone and number of docked presynaptic vesicles). However, the samples must be very thin, requiring complex preparation to avoid artifacts, and the images obtained are two-dimensional (2D) projections of the sample (micrographs). By contrast, SEMs scan the surface of the sample with a focused beam of electrons to provide a three-dimensional (3D) image, which can be used to study synapse morphology or measure dendritic spine volume and density within a brain region (Borczyk et al., 2019). However, the resolution of SEM (∼0.5 nm) is lower than TEM.

Based on the characteristics of these microscopes, variations have been developed to overcome their limitations (sample preparation, resolution, and 2D vs. 3D acquisition) and to allow combination with other techniques. Some examples of these derived methods are electron tomography (which uses TEM to generate a high-resolution 3D image), immuno-EM [which combines the use of gold-labeled antibodies with EM (D’Amico and Skarmoutsou, 2008; Liu et al., 2019)], array tomography [which combines the use of immunofluorescence (IF) with SEM to depict the specific components of the synapse (Micheva and Smith, 2007; Collman et al., 2015)], and focused ion beam milling-SEM (FIB-SEM, which allows the actual quantification of the density of synapses in a brain area through the automatization of the steps of sectioning and image acquisition (Merchan-Pérez, 2009; Santuy et al., 2020).

#### Electron Microscopy in Preclinical Research

In preclinical research, EM has mainly been applied to map synaptic structures and regional densities in the brain of healthy wild type (WT) animals (Kaplan and Hinds, 1977; Harris and Weinberg, 2012; Santuy et al., 2018). Several studies have also explored the concept of *synaptic plasticity* through different paradigms such as: (1) evaluating how the PSD and dendritic spines modify their shape and structure after a chemically induced long-term potentiation (Borczyk et al., 2019), (2) studying synaptic reorganization and reactive synaptogenesis/synaptic loss in response to a lesion (DiFiglia et al., 1988; Marrone et al., 2004; Kim and Jones, 2010; Li Q. et al., 2018), or (3) analyzing the effect of maturation and aging in the synapse (Kaplan and Hinds, 1977; Harris and Weinberg, 2012; Fan et al., 2018; Santuy et al., 2018). These studies emphasize both the existence of synaptic remodeling (even during adulthood) and the presence of compensatory mechanisms for a decrease in synaptic density (such as the increase in the size and surface area of the remaining connections) (Scheff et al., 1991; Dawirs et al., 1992; Calì et al., 2018).

The use of EM to study synapses in the context of neurodevelopmental and neurodegenerative animal models remains limited, partially due to its spatial restrictions as well as laborious and lengthy analytical methods. Predominately, studies have focused on understanding changes in synaptic density in relation to AD, in mouse models such as Tg2576 (AD model expressing human mutant form of the amyloid precursor protein, APP). This research confirmed the expected decrease in synapses in regions known to be affected by AD pathology — such as the hippocampal dentate gyrus or the entorhinal cortex (Dong et al., 2007) — and showed improvements in synaptic deficits after experimental treatments (Pérez-González et al., 2014; Xiao et al., 2020). Most importantly, these findings served either to clarify previous inconsistencies regarding synaptic loss measured with other methods (such as synaptophysin IHC (Dong et al., 2007)) or to corroborate results obtained by using different techniques (Pérez-González et al., 2014; Fan et al., 2018).

Several other neurodevelopmental and neurodegenerative disease models have shown alterations in synaptic density. These include, for example, a decrease in the synapse-to-neuron ratio in the Ts65Dn mouse model of Down syndrome (Ayberk Kurt et al., 2004), an absence of life-long changes in total synaptic density (number of synapses and synapses onto spines) in the R6/2 mouse model of Huntington’s disease (Savage et al., 2020), and a decrease in spine density and ultrastructural spine abnormalities in the A53T-BAC-*SNCA* mouse model of Parkinson’s disease (Parajuli et al., 2020).

#### Limitations

Despite the multiple possibilities of EM for the study synaptic density, its use in clinical and preclinical research is restricted due to the *ex vivo* nature of this technique, the high costs of purchasing and maintaining electron microscopes, and the complexity and skillset required for the data acquisition and analysis.

Furthermore, the brain tissue must be carefully processed, which typically involves the use of specific aldehyde fixatives and chemical compounds to preserve the ultra-structure of the synapse. Subsequently, the tissue is embedded in a resin to be sectioned in ultrathin sections (<150 nm) using an ultramicrotome. As these treatments themselves can induce artifacts or changes in the synaptic structure (Siksou et al., 2009), specialist training is required. Because of these barriers, the applications of EM and its combination with other *ex vivo* techniques remain limited.

Finally, the magnification power of electron microscopes comes with a trade-off of reduced field of view and sample size, hampering both global and multi-regional analyses of the brain. Nevertheless, recent improvements in EM methods for imaging large volumes may soon facilitate the visualization of larger brain areas (Kasthuri et al., 2015) or even the whole brain (Mikula et al., 2012).

### Histology and Immunohistochemistry

While EM is arguably the best and most direct method to study synapses *ex vivo*, histology and IHC are the most common, owing to their wider accessibility, ease of use and lower cost. These techniques are employed with two main purposes: to evaluate the morphology and density of dendritic spines — present predominantly, but not exclusively, in excitatory synapses (Scheuss and Bonhoeffer, 2014) — and to detect changes in the expression of pre- and postsynaptic proteins with specific antibodies.

Histological quantification of dendritic spine morphology and density has been of much interest in the study of normal and pathological brain conditions, due to the important role of these structures in most excitatory synapses (Nimchinsky et al., 2002); their involvement in synaptic plasticity, learning and memory processes (Mahmmoud et al., 2015; Ma and Zuo, 2021); and the presence of abnormal spines in many brain disorders (Fiala et al., 2002; Blanpied and Ehlers, 2004; Herms and Dorostkar, 2016). The quantification of spines — the number of (visible) spines per micrometer of dendrite — is considered to represent an index of synaptic density and functionality, due to the presence of these structures in most synapses. Recent research has highlighted the existence of a bidirectional relationship between spine maturation/functionality and synapse stability. For example, it was reported that even subtle changes in spine structure or motility can affect synaptic transmission (Wong, 2005; Lordkipanidze et al., 2013; Obashi et al., 2021), and that an increase in synaptic strength promotes the emergence and maturation of spines (Hotulainen and Hoogenraad, 2010; Fortin et al., 2012).

Among the *ex vivo* methods used to visualize changes in spine morphology and density in brain sections, Golgi staining is the oldest and most commonly used. This impregnation method involves the accumulation of heavy metal ions (silver or mercury) on the surface of a small subset of neurons, allowing the visualization of their entire structure, including the dendritic spines. Since its inception, this method has been improved (Pilati et al., 2008), modified to make it faster (e.g., Golgi-Cox method (Ranjan and Mallick, 2010)), and even combined with other techniques such as EM (Fairén, 2005) or tissue clearing and fluorescence (Vints et al., 2019). Despite the relevance and utility of Golgi staining, it also presents disadvantages such as random and unpredictable cell staining (Mancuso et al., 2013). Therefore, alternative methods have been developed, such as staining with the carbocyanine dye DiI — a fluorescent dye whose insertion into the lipid membrane of the neurons allows the visualization of their architecture (Gan et al., 2000; Cheng et al., 2014; Ba̧czyńska et al., 2021) — or the use of Lucifer Yellow or *XFP* [green (GFP), yellow (YFP), cyan (CFP) and red (RFP)] fluorescent proteins that label the entire neuronal structure (Feng et al., 2000). All these techniques allow quantification of potential changes in spine morphology and density when combined with manual and automatic analysis of maximum intensity projection (2D images) or 3D and even 4D image reconstruction (Dickstein et al., 2016; Basu et al., 2018; Kashiwagi et al., 2019; Ba̧czyńska et al., 2021). Moreover, the *in vivo* visualization of spine dynamics is possible in freely moving animals, thanks to the combination of transgenic mice expressing genetically tagged fluorescent proteins (such as PSD-95:GFP, or Thy1-YFP mice) and new microscopy methods (such as two-photon microscopy) (Niell et al., 2004; Yang et al., 2014; Garré et al., 2017).

Immunohistochemistry and particularly IF (which involves the use of a secondary antibody chemically conjugated to a fluorescent dye) has been extensively used to evaluate changes in the expression of pre- and postsynaptic proteins as indices of synaptic density. One of the advantages of this technique is the possibility of differentiating between excitatory and inhibitory synapses, owing to the availability of multiple antibodies that target proteins predominantly expressed in these synapses (e.g., excitatory synapses express PSD-95 whereas inhibitory synapses express gephyrin) (van Spronsen and Hoogenraad, 2010; Sheng and Kim, 2011; McLeod et al., 2017; Favuzzi and Rico, 2018). Such differentiation of targets has been an important tool in the study of selective gains and losses of synapses in diseases such as AD (Lauterborn et al., 2021). Moreover, the ability to distinguish between synaptic proteins has also led to the discovery that PSD-95 and neuroligin-1 are among the key regulators of the ratio between the number of excitatory and inhibitory synapses in the brain, the imbalance of which has been hypothesized as an underpinning factor of some brain disorders (Prange et al., 2004; Keith, 2008). In contrast, most presynaptic proteins, including SV2A and synaptophysin, are expressed in both excitatory and inhibitory synapses, allowing a global quantification of synaptic density. Such quantification is mostly performed by first applying a background correction and then analyzing the distribution, number, or intensity of the immunoreactive puncta (Ippolito and Eroglu, 2010; McLeod et al., 2017; Guirado et al., 2018), or evaluating the mean fluorescence intensity within a region (Shihan et al., 2021).

A summary of the synaptic proteins most commonly used in the analysis of synaptic density is shown in Table 1.

**TABLE 1:** Main pre- and post-synaptic proteins used as synaptic density markers.

Localization		Proteins	Role in synapse	Present in…	References
*PRESYNAPSE*	Vesicle	vGLUT (1–3)	Vesicular storage – glutamate	Excitatory synapses*, astrocytes, microglia	Fremeau et al., 2004; Martineau et al., 2017
		vGAT	Vesicular storage – GABA/glycine	Inhibitory synapses*	Chaudhry et al., 1998; Saito et al., 2010
		Synapsin1, 2	Regulates the number of SVs available	All synapses, astrocytes	Bogen et al., 2009; Cesca et al., 2010
		Synaptophysin-1 (a, b)	Regulates endocytosis	All synapses, astrocytes	Kwon and Chapman, 2011; Verstraelen et al., 2020
		Synaptotagmin-1	Calcium sensor – regulates exocytosis	All synapses, astrocytes	Yao et al., 2010; Bragina et al., 2012; Kiessling et al., 2018
		SV2 (A)	Regulates exo- and endocytosis	All synapses	Bartholome et al., 2017; Heurling et al., 2019
	Active zone	Bassoon	Assembly and organization of active zone with Piccolo	All synapses	Dieck et al., 1998; Waites et al., 2013; Gundelfinger et al., 2016
	Vesicle fusion machinery	SNAP-25	Vesicle fusion, calcium regulation, member of SNARE	All synapses, astrocytes	Irfan et al., 2019; Urbina and Gupton, 2020
		Syntaxin-1	Vesicle fusion, member of SNARE	All synapses, astrocytes	Vardar et al., 2016; Urbina and Gupton, 2020
		vAMP	Vesicle fusion, member SNARE	All synapses, astrocytes	Urbina and Gupton, 2020
	Adhesion	Neurexin	Formation/differentiation synapses	All synapses	Südhof, 2008, 2017
*POSTSYNAPSE*	Adhesion	Neuroligin (1–4)	Formation/maintenance synapses	1,3,4 in excitatory vs. 2 in inhibitory synapses	Südhof, 2008; Hu et al., 2015
	Scaffold	SNAP-25	Postsynaptic receptor trafficking, spine morphogenesis, and plasticity	Excitatory synapses	Antonucci et al., 2016; Hussain et al., 2019
		PSD-95 (a, b)	Regulates postsynaptic localization of excitatory receptors	Excitatory synapses	de Bartolomeis and Fiore, 2004; Keith, 2008
		Homer (1–3)	Synaptogenesis	Excitatory synapses	Sala, 2005; Verpelli et al., 2012
		Shank (1–3)	Synaptogenesis, spine maturation	Excitatory synapses	Sala, 2005; Monteiro and Feng, 2017
		Gephyrin	Brings and stabilizes inhibitory receptors at the postsynapse	Inhibitory synapses	Choii and Ko, 2015

SVs, synaptic vesicles; SNARE, synaptosomal-associated (SNAP) receptor. *vGLUT and vGAT can coexist in some excitatory and inhibitory synapses (Zander et al., 2010).

#### Histology and Immunohistochemistry in Preclinical Research

Histological examination of synapses in preclinical research has hitherto contributed to many important scientific discoveries in animal models of different brain disorders. As an example, abnormal spine morphology, density, and function have been related to cognitive deficits and reported in neurodevelopmental disorders such as Down syndrome or autism (von Bohlen und Halbach, 2010; Varghese et al., 2017; Torres et al., 2018). For instance, the Ts65Dn mouse model of Down syndrome exhibits a decrease in spine density but an enlargement of these structures, which seems to correlate with the severity of cognitive impairment (Belichenko et al., 2004, 2007). In autism models, mutations in genes coding for postsynaptic proteins such as *Shank3* result in modifications of dendritic spine morphology, suggesting a relationship between synaptic protein expression and spine morphology (Durand et al., 2012; Jiang and Ehlers, 2013; Zatkova et al., 2016; Guo et al., 2019).

However, not all the alterations in dendritic spines and synaptic protein concentration are associated with genetic factors: some of them are the result of internal changes (e.g., alterations in proteins such as Reelin), learning processes or environmental changes such as stress and isolation (Lieshoff and Bischof, 2003; Murmu et al., 2006; Bosch et al., 2016). Therefore, changes in spine density/morphology and in concentrations of different pre- and postsynaptic proteins have been extensively studied as markers and potential therapeutic targets of diverse pathologies and disorders (de Bartolomeis and Fiore, 2004; Dorostkar et al., 2015; Duman and Duman, 2015; Herms and Dorostkar, 2016; Ba̧czyńska et al., 2021).

##### Histology and Immunohistochemistry in Animal Models of Epilepsy

Epilepsy is characterized by the presence of uncontrolled and recurrent seizures, which are bursts of electrical neuronal activity. As most of this excitatory activity affects the dendritic spines, multiple research groups have studied the relationship between seizures and changes in the morphology and density of these structures (Wong and Guo, 2013; Tiwari et al., 2020; Xie et al., 2020). Specifically, spine density of the hippocampal and cortical pyramidal neurons appears to be reduced immediately after acute seizures, whereas it is recovered during the transition phase (period without seizures) and reduced, again, during the later chronic phase (period with recurrent spontaneous seizures) (Isokawa, 1998; Guo et al., 2012; Xie et al., 2020).

Interestingly, this alteration in spine density seems to precede neuronal death and has been suggested as a potential reason behind the cognitive and memory deficits observed in this disease (Wong, 2005). However, the results of combining *in vivo* imaging (two-photon microscopy) of dendritic spines with electroencephalography (EEG) during focal neocortical seizures suggest that the presence of electrographic seizures only creates a predisposition toward, but not necessarily causes, dendritic spine degeneration (Rensing et al., 2005).

**FIGURE 3:**
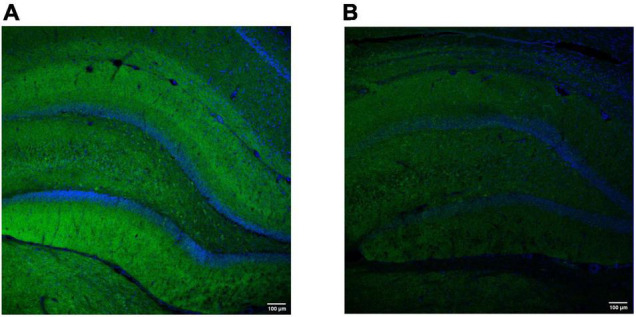
Representative hippocampal labeling of the synaptic vesicle protein synaptotagmin-1 in a control (A) and (B) epilepsy [kainic acid rat model of temporal lobe epilepsy (Levesque and Avoli, 2013)]. Both images were obtained using the polyclonal rabbit anti-Syt1 (Abcam, Cambridge, MA; Cat#ab131551); dilution 1:100 (overnight, 4°C). The secondary antibody was donkey anti-Rabbit Alexa Fluor488-conjugated (Thermo Fisher Scientific, Oregon, United States; Cat#A-21206); dilution 1:500 (45 min, RT). DAPI was used to counterstain (blue). The images were obtained with a scanning laser microscope (Leica TCS SP5 with AOBS, Leica Microsystems IR GmbH, Germany) with a 20 × magnification and similar exposure time. Note a decrease in synaptotagmin-1 labeling in the epileptic rat (3 months after kainic acid administration), compared to the control. Images were obtained at GIGA-CRC *in vivo* imaging and the GIGA-Imaging platform, ULiège (Belgium).

Another way to study changes in synaptic density during epileptogenesis is to quantify synaptic proteins such as SV2A, synaptophysin or synaptotagmin (see Figure 3). Indeed, these proteins are mostly decreased in preclinical models of epilepsy, which could be reversed by pharmacological treatments or by exposure to an enriched environment (van Vliet et al., 2009; Hanaya et al., 2012; Salaka et al., 2021), confirming the aforementioned utility of synaptic IHC as biomarker of epilepsy. On the other hand, reports of increases in the expression of the same markers (Contreras-García et al., 2018; Wang et al., 2018), highlight the possible presence of compensatory mechanisms including, for example, an increase in GABAergic neurotransmission (Ohno et al., 2012).

##### Histology and Immunohistochemistry in Animal Models of Alzheimer’s Disease

Changes in the morphology and density of dendritic spines have been postulated as main reasons for the synaptic and neuronal loss observed in AD. Therefore, dendritic spines are promising targets for new treatments (Knobloch and Mansuy, 2008; O’Neal et al., 2018; Ettcheto et al., 2020). Pathological extracellular deposits of amyloid-betaqq (A) protein, or Aβ plaques, seem to have a major role in the aberrant spine morphology and decrease in spine density observed in AD models (Dorostkar et al., 2015; Marttinen et al., 2018). Interestingly, A plaques were associated with elevated activity of calcineurin, which affects the morphology and density of dendritic spines through the inhibition of peptidyl-prolyl isomerase Pin1 signaling (Stallings et al., 2018; Reza-Zaldivar et al., 2020). This led to O’Neal et al. (2018) suggesting that an FDA approved calcineurin inhibitor be tested in AD patients. However, evidence from the Tg2576 mouse model of AD suggests that early decreases in spine density correlate with cognitive impairment and emerge before any measurable accumulation of insoluble A protein (Jacobsen et al., 2006).

Measurements of pre- and postsynaptic proteins have also been used as markers of synaptic density in AD models. Such investigations led to the discovery, in the 2xTg (APP/PS1) mouse, of an association between reduced SV2A and gephyrin in the nucleus accumbens (NAc), increased intracellular Aaccumulation, and decreased glycinergic (inhibitory) miniature synaptic currents (Fernández-Pérez et al., 2020).

##### Histology and Immunohistochemistry in Animal Models of Neuropsychiatric Disorders

Alterations in the morphology and density of dendritic spines, and in the expression of pre- and postsynaptic proteins have been associated not only with different neuropsychiatric disorders including depression and schizophrenia, but also with addiction to drugs of abuse such as psychostimulants (Robinson and Kolb, 1999; Blanpied and Ehlers, 2004).

In animal models of stress and depression, the reported changes in spine morphology and density seem to be strongly influenced by multiple parameters, including the model used, the sex and genetic strain of the animals, and the brain region analyzed (Murmu et al., 2006; Bock et al., 2011; Duman and Duman, 2015; Qiao et al., 2016). Most studies reported a decrease in spine density in the prefrontal cortex and hippocampus — regions typically atrophied in people suffering from depression and associated with the severity of the disease (Taylor et al., 2014)— but an increase in spine density in the amygdala and the NAc. Importantly, some of these alterations could be rescued by treatments with antidepressants such as ketamine (Krzystyniak et al., 2019; Ali et al., 2020). However, the existence of contradictory results — e.g., the absence of spine loss or even an increase in spine density in some animal models (Fox et al., 2020)— highlights the importance of creating standardized protocols and improving replicability between laboratories. Regarding the measurement of synaptic proteins, the evaluation of vesicular GABA transporter (vGAT) and gephyrin in mice that underwent chronic social defeat stress is more in agreement with the results observed in humans, where there was a significant decrease in both these synaptic markers in the NAc (Heshmati et al., 2020).

In schizophrenia, the research evaluating changes in dendritic spines is more consistent, with multiple laboratories reporting decreases in spine density and dendritic arborization in brain regions such as the primary visual cortex, the prefrontal cortex and the subiculum (Moyer et al., 2015; Tendilla-Beltrán et al., 2019; Chen P. et al., 2021). Despite some contradictory results, several studies have suggested that a reduction in microtubule-associated protein 2 (MAP2) — a constituent of the PSD — is one of the reasons for the observed spine density decrease (Gu et al., 2008; Jaworski et al., 2009). In agreement with the notion of decreased spine density in schizophrenia, lower levels of PSD-95 have also been found in the frontal cortex and ventral hippocampus of the sub-chronic phencyclidine (PCP) mouse model of schizophrenia (Gigg et al., 2020). A recent meta-analysis reviewed research quantifying multiple synaptic measures (dendritic spine density, PSD number and PSD protein expression levels) in post-mortem human brain tissue, from different methods (Golgi staining, IHC and EM) (Berdenis van Berlekom et al., 2020). The results of this meta-analysis are consistent with those obtained in preclinical research, with most studies highlighting a significant decrease in synaptic density in the brain of patients with schizophrenia compared to healthy controls.

#### Limitations

There are several aspects to consider when using histological and IHC techniques to quantify synaptic density. Some of these are related to the nature of the target. For instance, the evaluation of spine density should be considered only as a partial biomarker of synaptic density, since not all synapses are formed on dendritic spines, which are absent in most inhibitory interneurons (Markram et al., 2004). The quantification of pre- and postsynaptic proteins, commonly present in chemical synapses, also disregards the existence of electric synapses and their important role in brain development (Szabo, 2004; Todd et al., 2010) and in the adult nervous system (Connors and Long, 2004; Pereda, 2014), especially in local inhibitory circuits (Galarreta and Hestrin, 2001; Vervaeke et al., 2012). Furthermore, it is important to carefully select which synaptic protein will be used as a synaptic density marker, since they are not only disease-specific but also have differential expressions across time and brain regions. For example, there is a selective, regional protein loss in AD patients, with some hippocampal postsynaptic proteins being less affected by the disease, while others present even an increase in their expression (Clare et al., 2010, de Wilde et al., 2016). Additionally, multiple studies have highlighted the presence of presynaptic proteins not only in neurons, but also in astrocytes. For instance, cultured astrocytes seem to express proteins such as synaptobrevin-2 (the so-called vesicle-associated membrane protein 2 or vAMP2), synaptotagmin-1, synaptophysin and SNAP-25 (Maienschein et al., 1999; Wilhelm et al., 2004; Singh et al., 2014), which is involved in the release (exocytosis) of glutamate-containing vesicles (Zhang et al., 2004; Crippa et al., 2006; Mielnicka and Michaluk, 2021). Recently, the vesicular glutamate transporter (vGLUT) has also been found in microglia (Brioschi et al., 2020). These studies highlight the importance of employing multiple antibodies (markers) and even different methods to measure synaptic structure and density before reaching any conclusion about how these are affected in neurological and neuropsychiatric disorders.

Other limitations of histology and IHC derive from the methodology itself: despite the existence of multiple antibodies to target pre- and postsynaptic proteins, some of them have poor labeling performance (e.g., lack of specificity and/or sensitivity) and, therefore, are not suitable to quantify synaptic density (Verstraelen et al., 2020). This specificity problem could explain the contradictory results obtained with some of these antibodies, which urges the development of new and more specific antibodies to precisely quantify synaptic density. In the case of IF, a bias in the measurement of synaptic density could also be introduced by the characteristics of the fluorophore and the fluorescence signal, which is expected to fade over time, precluding comparison of images obtained at different time points.

Finally, the intrinsic characteristics of the image acquisition and analysis are also a source of bias. In this regard, confocal laser-scanning microscopes often produce partial and distorted results due to their insufficient spatial resolution. A clear example is the bias in estimating spine density from 2D images, where many spines can be hidden from the field of view depending on their position on the dendrite. In recent years, the refinement of optical brain clearing techniques and tissue expansion-enabled imaging — extensively reviewed elsewhere (Gómez-Gaviro et al., 2020; Parra-Damas and Saura, 2020; Ueda et al., 2020) — have opened the door to a faster and more precise visualization of synapses, facilitating the study and the analysis of neural circuits even in deep brain structures (Hama et al., 2011) or living animals (Iijima et al., 2021). Additionally, the development of super-resolution fluorescence microscopy techniques — such as 3D stimulated emission depletion (STED) microscopy (Vicidomini et al., 2018; Sahl and Hell, 2019) or super-resolution shadow imaging (SUSHI) (Tønnesen et al., 2018)— has improved image resolution and decreased the presence of artifacts, enabling a more precise assessment of alterations in spine structure and density. However, these methods are not yet widely accessible and still require manual or semi-manual processing of images due to the absence of sufficient 3D data to properly implement machine learning algorithms (Ruszczycki et al., 2012; Belthangady and Royer, 2019).

### Other Post-mortem Methods: Autoradiography

**FIGURE 4:**
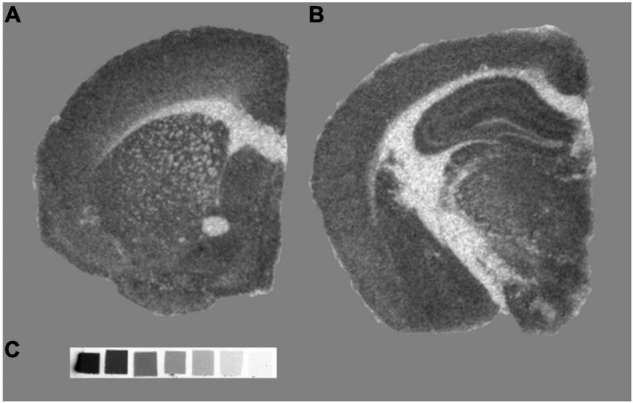
SV2A autoradiography with [^3^H]UCB-J, performed in an adult C57BL/6 mouse. (A,B) Two representative autoradiographs showing [^3^H]UCB-J labeling. (C) ^3^H standard for quantification (ART-123A American Radiolabeled Chemicals Inc., United States). Slices (20 μm) were mounted onto a glass slide (Superfrost™) and incubated with 3 nM [^3^H]UCB-J (Novandi Chemistry AB, Sweden). Once dried, the slide was placed into light-tight cassettes with the radioactive standard slide and a hyperfilm (Amersham 8 × 10 in Hyperfilm Scientific Laboratory Supplies, United Kingdom). Films were exposed for 2 weeks before being developed in a Protex Ecomax film developer (Protec GmbH & Co, Germany). Images acquired at BRAIN Centre, King’s College London, London, United Kingdom.

Imaging synaptic density in post-mortem tissue is also possible with autoradiography, which involves imaging the distribution of molecules labeled with radioisotopes [e.g., hydrogen-3 (^3^H) or iodine-125 (^125^I)] in tissue sections (see Figure 4).

This technique presents two variations: *in vitro* autoradiography, which involves incubating mounted tissue with a radiolabeled ligand, and *ex vivo* autoradiography, which involves injecting a radiolabeled ligand at tracer concentrations (hence *radiotracer*) into a living animal before collecting, cutting, and mounting the tissue. In both forms, images of the radioligand distribution are acquired from the brains processed post-mortem (Maurer, 1984; Ishiwata et al., 1999; Griem-Krey et al., 2019). Autoradiography has proved useful for characterizing newly developed positron emission tomography (PET) tracers, due to its simplicity and relatively low cost, providing information about the metabolism of the radiotracers, ligand selectivity and target localization (Solon, 2012; Manuel et al., 2015; Griem-Krey et al., 2019). Furthermore, autoradiography provides higher spatial resolution images than PET (μm vs. mm), which enables the quantification and localization of binding in small anatomical structures of rodent brains (Schmidt and Smith, 2005). However, this method also has some limitations, such as the unsuitability for longitudinal studies, or the limited information that it provides about radiotracer kinetics (Kuhar and Unnerstall, 1985; Schmidt and Smith, 2005).

The use of autoradiography for quantification of synaptic density in preclinical research has been made possible by the development of SV2A radiotracers, described in more detail in the following section. These radiotracers have been used to map the expression of the presynaptic SV2A protein in the brain of WT and transgenic animals (Menten-Dedoyart et al., 2016; Varnäs et al., 2020), to evaluate changes in synaptic density in the 6-hydroxydopamine (6-OHDA) model of Parkinson’s disease and the quinolinic acid model of Huntington’s disease (Thomsen et al., 2021), and to study the effect of different drugs and treatments (Onwordi et al., 2020; Binda et al., 2021; Halff et al., 2021; Raval et al., 2021b).

## Measuring Synaptic Density *in vivo*

Even though the spatial resolution of *in vivo* techniques is significantly poorer than that of *ex vivo* methods, measuring synaptic density *in vivo* presents many advantages, such as the possibility of quantifying the number of synapses longitudinally, which may result in earlier diagnosis of brain disorders. This characteristic has recently promoted the use of these techniques as important biomarkers in neurodegenerative and psychiatric disorders.

In this section we cover the most used *in vivo* techniques, which explore two different aspects of the synapse: the concentration of the SV2A protein (SV2A PET tracers), and the concentration of glutamate (gluCEST).

### Assessing Synaptic Density by Positron Emission Tomography

The last decade has seen a significant rise in the synthesis and use of SV2A PET tracers. These radioactive compounds are commonly designated as *synaptic density radiotracers* due to their affinity and specificity for the SV2A protein: a transmembrane presynaptic protein present in synaptic vesicles that plays an important role in synaptic transmission — specifically in the calcium-dependent release of neurotransmitters (through its interaction with synaptotagmin-1) — and in synaptic vesicle recycling (interacting with synaptotagmin-1 and stonin-2) (Mendoza-Torreblanca et al., 2013; Bartholome et al., 2017). Traditionally, this protein has been associated with epilepsy for three main reasons: (1) SV2A is the molecular target of the antiepileptic drug levetiracetam, (2) SV2A knockout mice suffer from seizures from postnatal day 7 and die 2 weeks later, and (3) the brain expression of SV2A in animal models and patients with epilepsy is lower than in healthy controls (Crowder et al., 1999; Lynch et al., 2004; Klitgaard and Verdru, 2007; van Vliet et al., 2009; Sills, 2010).

The discovery of the relationship between SV2A and epilepsy triggered the development of the first SV2A radiotracer, [^3^H]ucb 30889, with the aim to identify levetiracetam’s binding sites in the rat brain and spinal cord (Gillard et al., 2003; Lambeng et al., 2005). Despite its utility, the extremely long half-life (>12 years) of tritium precluded the use of this radiotracer in humans. In 2013, the creation of the first PET-suitable radiotracer, [^18^F]UCB-H, allowed the *in vivo* measurement of changes in SV2A brain levels in both humans and animals (Bretin et al., 2013; Warnock et al., 2014; Becker et al., 2017; Serrano et al., 2018). Since then, other PET radiotracers have been developed, such as [^11^C]levetiracetam (Cai et al., 2014), [^11^C]UCB-A (Estrada et al., 2016), and [^11^C]UCB-J (Nabulsi et al., 2016). The latter, [^11^C]UCB-J, displays higher specific binding capacity than [^18^F]UCB-H, with also a high brain uptake, fast *in vivo* kinetics and rapid metabolism. Nevertheless, the half-life of carbon-11 (^11^C, 20.4 min) is significantly shorter than that of fluorine-18 (^18^F, 109.8 min), which precludes the routine use of [^11^C]UCB-J as it requires an on-site cyclotron for its synthesis. New radiotracers, such as [^18^F]SynVesT-1 — previously called [^18^F]SDM-8 and [^18^F]MNI-1126 — and [^18^F]SynVesT-2 (also named [^18^F]SDM-2) are supposed to overcome this problem by combining the specificity for SV2A and the fast *in vivo* binding kinetics of [^11^C]UCB-J with the longer half-life of [^18^F]UCB-H (Li S. et al., 2018; Constantinescu et al., 2019; Cai et al., 2020; Patel et al., 2020; Sadasivam et al., 2021).

The development of these novel SV2A PET tracers occurred in parallel with the discovery of the important role of SV2A in the onset and development of multiple neurological and neuropsychiatric disorders, opening the door to a wide spectrum of applications (Mattheisen et al., 2012; Stockburger et al., 2015; Cortès-Saladelafont et al., 2016; Li and Kavalali, 2017; Heurling et al., 2019). Nevertheless, it was not until 2016 that the full potential of these radiotracers was unveiled: the possibility to detect changes in synaptic density *in vivo* (Finnema et al., 2016; Morris, 2016; Mercier et al., 2017) through regional quantification of brain SV2A expression.

#### Synaptic Vesicle 2A Positron Emission Tomography Tracers in Preclinical Research

The use of SV2A PET tracers in preclinical research has been key to improving their synthesis, specificity, and kinetics, as well as to enable the *in vivo* quantification of synaptic density in a wide spectrum of neurological and neuropsychiatric disorders (Rabiner, 2018; Cai et al., 2019; Constantinescu et al., 2019). For example, (Serrano et al., 2020) explored changes in synaptic density at different time-points of the rat lifespan (late puberty to adulthood) evaluating in parallel how epileptogenesis affects the brain. This study showed an increase in synaptic density throughout the lifespan of healthy animals, in line with the recently reported increases in gray and white matter volumes (MacNicol et al., 2022). These results support the idea of brain plasticity in which synapses are continuously being formed and strengthened, highlighting the potential of quantifying SV2A *in vivo* to detect aberrancies in brain development.

In the following subsections we give an overview of the use of SV2A PET tracers in preclinical research. Given the relative novelty of some of these tracers, relatively few preclinical studies have been published to date. Furthermore, SV2A PET tracers are increasingly used in clinical research, where they have been successfully used to map out synaptic changes in AD and Progressive Supranuclear Palsy (Passamonti et al., 2017; Holland et al., 2020, 2021; O’Dell et al., 2021; Tuncel et al., 2021) as well as in other conditions such as depression (Holmes et al., 2019), schizophrenia (Onwordi et al., 2020), cannabis use disorder (D’Souza et al., 2020), and human immunodeficiency virus (HIV) (Weiss et al., 2021).

##### Synaptic Vesicle 2A Positron Emission Tomography Tracers in Animal Models of Epilepsy

Even though the development of SV2A tracers was motivated by epilepsy research, only three articles have been published to date in this area: two clinical proof-of-concept studies about the ability of [^11^C]UCB-J radiotracer to detect a decrease in synaptic density in patients with temporal lobe epilepsy and unilateral mesial temporal sclerosis (Finnema et al., 2016, 2020), and one preclinical study exploring *in vivo* changes in SV2A during the development of temporal lobe epilepsy (Serrano et al., 2020). The latter observed a progressive and region-dependent decrease in [^18^F]UCB-H binding (and, by extension, a decrease in synaptic density) in epileptic animals, with significant differences between groups even before the onset of seizures. Furthermore, the results obtained *in vivo* during the chronic phase of epilepsy were confirmed *ex vivo* with SV2A IF, highlighting not only the utility of SV2A PET as an epilepsy biomarker, but also the reliability of this *in vivo* technique.

##### Synaptic Vesicle 2A Positron Emission Tomography Tracers in Animal Models of Alzheimer’s Disease

[^11^C]UCB-J and [^18^F]SynVesT-1 have been used to study synaptic loss in two AD mouse models, ArcSwe and APP/PS1 (Sadasivam et al., 2021; Xiong et al., 2021), as well as to evaluate the potential effect of treating AD pathology with a Fyn kinase inhibitor, saracatinib (Toyonaga et al., 2019). Even though the methods employed to analyze radiotracer binding are different in these studies, they all reach the conclusion that there is a significant decrease in synaptic density in the hippocampus of AD animals, which can be rescued with saracatinib, and measured *in vivo* with SV2A PET radiotracers.

##### Synaptic Vesicle 2A Positron Emission Tomography Tracers in Animal Models of Neuropsychiatric Disorders

In psychiatric research, [^11^C]UCB-J has been used to measure synaptic density deficits in the *Sapap3* knockout model of obsessive-compulsive disorder, through a longitudinal PET study in which an early decrease in radiotracer binding was reported in several brain regions of this model, including in the cortex, striatum, thalamus, and hippocampus (Glorie et al., 2020).

#### Limitations

A general limitation of PET imaging is the difficulty in quantifying radiotracer binding, which usually requires drawing multiple blood samples at precise time-points to calculate the concentration of non-metabolized radiotracer in arterial plasma (arterial input function) (Laruelle, 2002; Acton et al., 2004). Even though non-invasive alternatives have been proposed — such as deriving the input function from the image, or the use of a reference region with no specific uptake — these methods also present associated problems, and their accuracy must be assessed before their routine use (Tomasi et al., 2012; Lammertsma, 2017; Serrano et al., 2018). With respect to the use of SV2A radiotracers, their results (although promising) must be taken with caution since they rely on three aspects: (1) the reliability of using SV2A as synaptic density marker in a specific disease, (2) the variability of SV2A expression due to external stimulus, and (3) the ability of the radiotracer to bind to the SV2A protein.

Regarding the first aspect, a selective loss and gain of different synaptic proteins has been reported in AD (Sze et al., 2000; de Wilde et al., 2016), with some contradictory results about changes in SV2A. For instance, several authors have reported unchanged SV2A levels in the middle frontal gyrus (Metaxas et al., 2019), the hippocampus, entorhinal cortex, caudate nucleus, and occipital cortex (Sze et al., 2000) in post-mortem samples from AD patients. In contrast, the *in vivo* comparison of healthy volunteers and AD patients with [^11^C]UCB-J PET showed a 41% reduction in hippocampal binding in the AD group (Chen M.K. et al., 2018). Interestingly, other synaptic proteins show a differential expression in AD, with the presynaptic proteins being more affected by the disease than the postsynaptic ones (de Wilde et al., 2016). These studies highlight the necessity of combining *ex vivo* and *in vivo* techniques and measuring different pre- and postsynaptic proteins to obtain a more reliable measure of synaptic density in a specific disease.

Concerning the second aspect, although SV2A expression is constant inside synaptic vesicles and presents a small intervesicle variation (Mutch et al., 2011), the number of synaptic vesicles in the presynapse is closely related to synaptic activity and functionality (Valtorta et al., 1990; Rizzoli and Betz, 2005). The number of synaptic vesicles, therefore, is expected to increase in the presence of a stimulus, raising the question whether the binding of SV2A radiotracers provides a stable measure of synaptic density or primarily reflects brain activity in the moment of scanning. This question has been recently tackled by Smart et al. (2021) where the effect of stimulating the visual cortex on the [^11^C]UCB-J binding was assessed through the measurement and comparison of three kinetic parameters: tissue influx (*K*_1_), volume of distribution (*V*_*T*_) and binding potential (*BP*_*ND*_). The results highlighted the stability of *V*_*T*_ and *BP*_*ND*_ during cortical stimulation and corroborated their utility as *in vivo* markers of SV2A levels and, for instance, as potential markers of synaptic density. On the contrary, *K*_1_ values increased during the visual stimulation and were significantly correlated with cerebral blood flow and fMRI BOLD signal assessed with the same paradigm. *K*_1_, therefore, reflects the radiotracer influx and should be considered an index of synaptic function rather than a synaptic density measure. In addition to the presence of a stimulus during the scan, a recent unpublished study (Miranda et al., 2021) has also suggested an effect of anesthesia on the measure provided by SV2A PET tracers. Specifically, the use of prolonged isoflurane anesthesia seems to produce a significant increase in [^18^F]SynVesT-1 uptake, compared with quickly anesthetized or awake animals. These results highlight the importance of maintaining similar and stable conditions between and within subjects to avoid potential bias in the quantification of SV2A levels.

Finally, the availability, conformation, and electrostatic properties of SV2A vary across the exocytosis process (Lynch et al., 2008; Shi et al., 2011; Correa-Basurto et al., 2015). Further studies should be carried out to determine how these changes affect the ability of the different SV2A PET radiotracers to recognize and bind to this presynaptic protein.

### Assessing Synaptic Density by MRI

While PET is the gold standard for *in vivo* molecular and metabolic imaging, it has the disadvantages of ionizing radiation, limited accessibility, and high cost. There are several MRI methods that provide cheaper, less invasive alternatives. For example, structural MRI can measure changes in cortical thickness as well as whole brain morphometry and regional deformations resulting from abnormal development or disease. However, structural MRI is neither specific nor direct and it does not have a resolution high enough to detect synapses *per se*, although some authors have attempted to connect regional variation of brain structures by MRI to the underlying synaptic spine densities (Keifer et al., 2015) or to plastic changes related to synaptic remodeling (Lerch et al., 2017). MR-based molecular imaging methods that can measure glutamatergic function may provide more promising indices of synaptic density.

#### Magnetic Resonance Spectroscopy and Chemical Exchange Saturation Transfer

MRI most commonly involves imaging ^1^H nuclei (i.e., protons), which in biological tissue are predominately found in water molecules. In contrast, MR spectroscopy (MRS) is a technique that can detect and quantify concentrations of various metabolites: the different protons of each metabolite have slightly different resonant frequencies and thus produce different peaks in an MR spectrum. But because these metabolites are found in much lower abundance than water, they produce much lower signal, limiting the spatial and temporal resolution of MRS techniques.

Chemical exchange saturation transfer (CEST) MRI is a method that overcomes the problem of low metabolite concentrations by exploiting the phenomenon of chemical exchange, whereby the protons of certain molecules exchange with those of water. The sensitivity of CEST MRI is increased by up to two orders of magnitude compared to MRS via continuous magnetic saturation of metabolite protons and subsequent exchange with bulk water protons over a period of seconds. This enables spatial mapping of metabolite concentrations at resolutions comparable to conventional MR imaging techniques. The basic principles of CEST MRI are covered in greater detail in a review by Wu et al. (2016).

Among the different CEST MRI methods, glutamate CEST or GluCEST is sensitive to changes in levels of glutamate, the main excitatory neurotransmitter in the brain, which can be detected by targeting the amine proton at an offset frequency of 3 ppm (Cai et al., 2012).

#### MR Spectroscopy and GluCEST in Preclinical Research

Previous studies using EM have shown that the highest levels of glutamate in the rat hippocampus are in the axon terminals of excitatory neurons (Bramham et al., 1990) and that glutamate levels correlate strongly with synaptic vesicle density (r = 0.94) in rat spinocerebellar mossy fiber terminals (Ji et al., 1991). However, the relationship between glutamate levels and synaptic density is not simple and can be altered in pathological conditions. For instance, a recently published study (Onwordi et al., 2021) showed that the correlation between the glutamate-to-creatine ratio measured by MRS and the [^11^C]UCB-J distributed volume ratio is only significant in healthy volunteers (hippocampus and anterior cingulate cortex) and not in patients with schizophrenia.

**FIGURE 5:**
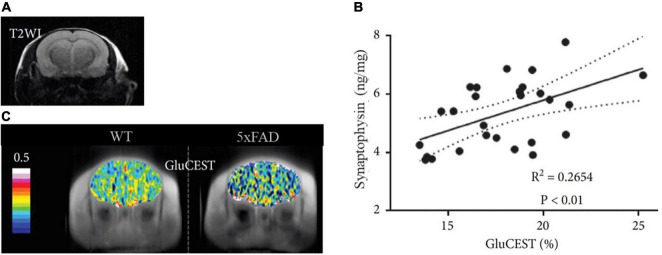
Representative images of GluCEST MRI of a 5xFAD and a WT mouse. (A) Structural T2-weighted image (T2WI) in the coronal plane. (B) GluCEST maps of the corresponding T2WI showing reduced GluCEST effects in an aged (7-month old) 5xFAD mouse compared with the WT. (C) Correlation between GluCEST and Synaptophysin concentration. Figure extracted and modified from Igarashi et al. (2020).

The relationship between glutamate levels and synaptic density has been further explored in animal models of neurodegeneration through the GluCEST sequence. For example, the GluCEST signal was associated with synaptophysin IHC in the PS19 mouse model of tauopathy, with both showing a decrease in the CA3 hippocampal region and the thalamus but not in the dentate gyrus or the entorhinal cortex (Crescenzi et al., 2014). GluCEST signal was also decreased in different mouse models of AD, such as the APP/PS1 and the 5xFAD models. In the APP/PS1 mouse, the GluCEST signal correlated with the glutamate-to-creatine ratio measured by MRS (Haris et al., 2013), while in the 5xFAD model, the decrease in glutamate was correlated with ELISA-based synaptophysin measurements [see Figure 5, extracted from Igarashi et al. (2020)]. This result suggests a relationship between the *in vivo* levels of glutamate and the *ex vivo* levels of synaptophysin, a presynaptic protein typically used as synaptic density marker.

#### Limitations

A technical limitation of GluCEST, as with many MRI methods, is its molecular specificity. Cai et al. estimated that 70–75% of the GluCEST signal comes from glutamate and the remaining 25–30% from creatine, GABA, and other molecules (Cai et al., 2012). More recently, a simulation study reported that glutamate contributes to about 60% of the GluCEST signal at 3.2 ppm at 7T and neutral pH, with this contribution increasing with decreasing pH and increasing field strength (Khlebnikov et al., 2019). Thus, the specificity of GluCEST can be maximized in preclinical studies conducted at ultra-high field strengths.

Additionally, the target selected to evaluate synaptic density is a limitation itself: the quantification of glutamate produces a bias in the measurement of synaptic density, since it only considers the number of glutamatergic synapses, disregarding the existence of other synapses in the brain, such as inhibitory or electric synapses. To date, and despite the ability of MRI to provide a measure of synaptic function (i.e., with functional MRI or fMRI), there is no other MRI sequence able to provide a better measure of synaptic density. Therefore, further improvements and different targets are necessary to obtain a reliable MRI biomarker of changes in synaptic density.

## Discussion

**FIGURE 6:**
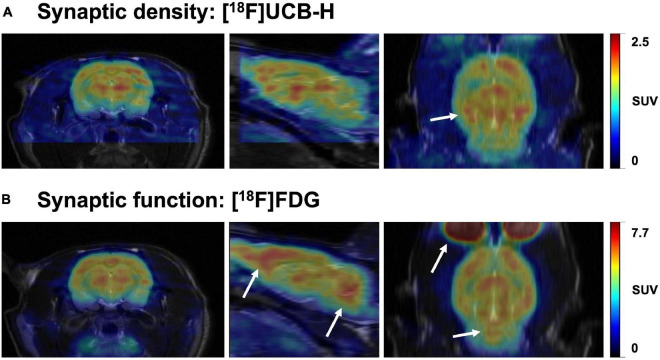
Representative brain images comparing synaptic density and function in the same rat. The images represent the uptake of two radiotracers: (A) [^18^F]UCB-H (concentration of SV2A protein, synaptic density marker) and (B) [^18^F]FDG (glucose metabolism, synaptic function marker). Both images were acquired 40-60 min after intravenous radiotracer injection (36 and 20 MBq, respectively). Scans were performed 24h apart, followed by a T2-weighted MR image to allow a better comparison between both PET scans through manual rigid-body co-registration with PMOD software. The white arrows represent the main differences in uptake between both radiotracers. While synaptic density and function are similar in multiple brain areas, regions such as the prefrontal cortex and cerebellum seem to have relatively higher glucose metabolism (synaptic function) than concentration of SV2A (synaptic density). Images obtained at GIGA-CRC *in vivo* imaging, ULiège (Belgium).

The brain changes constantly throughout the lifespan. The pursuit of understanding this plasticity has always been at the forefront of the neuroscientific community, considered as the path to neuroscience’s holy grail: to fathom the biological reason behind our singularity. In this quest, synaptic density is one of the gateways toward this goal, since the synapse is the functional unit of the brain (Mayford et al., 2012). Over the years, various *ex vivo* and *in vivo* methods have been developed to measure changes in synaptic density, structure, and function, each one of them targeting different aspects of the synapse. Importantly, although various studies often conflate synaptic density and synaptic function (Chen M.K. et al., 2021; Raval et al., 2021a; van Aalst et al., 2021), we must bear in mind that these two are different — although related — concepts that may sometimes lead to different results (see Figure 6). This distinction is also reflected in the methodology employed to quantify synaptic density, predominantly carried out in post-mortem tissue (e.g., EM and IHC), whereas synaptic function is by default linked to experiments in living tissues, using electrophysiology, electroencephalography, or brain imaging with [^18^F]FDG PET and fMRI. Moreover, synaptic density appears to be a more stable biomarker than synaptic function which, due to the nature of the target measured (i.e., electrical activity, glucose and blood oxygenation), can be affected by both internal and external stimuli, such as tissue preparation for *ex vivo* assessments (Kirov et al., 1999) or anesthesia used during PET or MRI scanning (Fueger et al., 2006; Aksenov et al., 2015; Spangler-Bickell et al., 2016; Paasonen et al., 2018; van der Linden and Hoehn, 2022), as well as physiological fluctuations during live animal imaging (Harris et al., 2018; Steiner et al., 2020).

In this review, we have briefly presented the most commonly used methods for quantifying synaptic density, focusing on their applications in preclinical studies of neurodevelopmental and neurodegenerative disorders. Even though many of these methods lead to the same conclusion — the existence of degeneration, synaptic density loss or dendritic spine abnormalities in models of neurological diseases — all of them also have limitations (e.g., resolution, specificity, sensitivity, and complexity) that should be considered when interpreting the results (see Table 2). To overcome some of these limitations, alternatives are being explored, such as the combination of existing techniques, or the development of brand-new methods to quantify synaptic density.

**TABLE 2:** Summary of main methods for imaging brain synaptic density.

Methodology		Synaptic target	Advantages	Shortcomings
*EX VIVO*	EM	Visualization of synapse: synaptic structure and synaptic density	Allows the actual visualization of the number of synapses Possibility to differentiate between inhibitory and excitatory synapses.	Expensive and time-consuming Requires a complex sample preparation, which can affect the results and limit the combined use of other *ex vivo* techniques
	
	Histology and IHC	Morphology and density of dendritic spines and expression of pre/post synaptic proteins	Cheap and accessible to all laboratories Possibility to differentiate between inhibitory and excitatory synapses	Some antibodies present specificity/sensitivity problems that can bias the results Not all synapses involve dendritic spines (e.g., electric synapses) and not all synaptic proteins are affected in all diseases

*IN VIVO*	SV2A PET TRACERS	Expression of the presynaptic protein SV2A	Allows the *in vivo* evaluation of synaptic density Validated to be used in both animals and humans, allowing translational research	Lower spatial resolution than the other methods and more difficult to quantify accurately Requires facilities adapted to work with radioactivity and the administration of a radiotracer It is not possible to differentiate between inhibitory and excitatory synapses
	
	MRI	Glutamate concentration	Higher spatial resolution than PET Does not require a pre-treatment or administration of a substance/drug.	Low specificity and sensitivity compared to PET Glutamate is not a good marker: it can also be found in astrocytes, and it does not account for inhibitory synapses

*EM, electron microscopy; IHC, immunohistochemistry; SV2A, synaptic vesicle 2A protein; PET, positron emission tomography; MRI, magnetic resonance imaging.*

With respect to the combined use of existing techniques, array tomography marries the *ex vivo* methods of IF and volumetric EM, enabling the quantification of precise molecular targets and the direct visualization of structural changes in synapses with unprecedented specificity and spatial resolution (Micheva and Smith, 2007; Prieto and Cotman, 2017). An example of combining *in vivo* methods is the use of PET and MRI techniques [e.g., (^11^C)UCB-J PET and fMRI], which may help shed light on the relationship between synaptic density and synaptic function. For example, a reduction in [^11^C]UCB-J binding has recently been associated with aberrant neural network function and inversely correlated with the depressive symptomatology in patients with major depressive disorder (Holmes et al., 2019).

Regarding the development of new methods to measure synaptic density, the use of genetically encoded fluorescent molecules as indicators of neuronal activity is particularly promising (Hamel et al., 2015; Lin and Schnitzer, 2016). These molecules, when examined with multiphoton microscopes (e.g., two-photon microscopy) (Lecoq et al., 2019), allow imaging of different processes involved in synaptic transmission in freely behaving animals, such as vesicle release (Sankaranarayanan et al., 2000; Ferro et al., 2017; Bensussen et al., 2020) or intracellular calcium dynamics (Chen et al., 2013; Ziv et al., 2013; Sheffield and Dombeck, 2015; Yang et al., 2016). These techniques, even though they offer the possibility of obtaining a high-resolution visualization of synaptic transmission elements, also suffer from limitations such as a narrow depth of field (1.2 mm) (Dunn and Sutton, 2008; Benninger and Piston, 2013). New technologies are quickly emerging to overcome these limitations, such as gradient-index (GRIN) lenses (Meng et al., 2019; Chien et al., 2021) and two-photon miniscopes (miniaturized head-mounted microscopes) (Ghosh et al., 2011; Silva, 2017; Zong et al., 2017, 2021). These technologies provide deeper and fast high-resolution volumetric images of dendrites and spines in freely moving animals, helping to better understand the functioning of the living brain.

Throughout this review we have explored different methods for quantifying synaptic density in preclinical research, focusing on their potential as biomarkers of neurological and psychiatric disorders. However, we should highlight the translatability of most of these methods, which are currently being used in clinical research. In this regard, EM and IHC have been extensively used to examine synaptic density in post-mortem human brain samples, with the purpose of mapping neuronal connectivity in healthy donors (Glantz et al., 2007; Kay et al., 2013; Lewis et al., 2019; Sherwood et al., 2020), or in the framework of understanding the pathology behind some of the disorders tackled in this review (Fourie et al., 2014; Osimo et al., 2019; Lauterborn et al., 2021). Furthermore, the recent emergence of multiple SV2A PET tracers has opened the door for the study of synaptic density in the living human brain, with exciting implications for clinical practice (Finnema et al., 2016). These radiotracers have already been used to explore changes in synaptic density in patients with epilepsy, AD, depression, schizophrenia, and other diseases, with promising results (Bao et al., 2017; Chen M.K. et al., 2018; Holmes et al., 2019; Finnema et al., 2020; Onwordi et al., 2020; Radhakrishnan et al., 2021). Therefore, although there are still some challenges to successfully employ synaptic density as a diagnostic and/or prognostic biomarker for neurological and neuropsychiatric disorders, current methods of measuring synaptic density can help us understand both healthy and disordered brain development and function.

## Author Contributions

MS, MP, and DC contributed to the conception and design of the review. MS and EK wrote the first draft of the manuscript. MP, FT, and DC contributed to manuscript editing and revision. All authors read and approved the submitted version.

## Conflict of Interest

The authors declare that the research was conducted in the absence of any commercial or financial relationships that could be construed as a potential conflict of interest.

## Publisher’s Note

All claims expressed in this article are solely those of the authors and do not necessarily represent those of their affiliated organizations, or those of the publisher, the editors and the reviewers. Any product that may be evaluated in this article, or claim that may be made by its manufacturer, is not guaranteed or endorsed by the publisher.
